# Ganglion Cell Complex Evaluation in Exudative Age-Related Macular Degeneration after Repeated Intravitreal Injections of Ranibizumab

**DOI:** 10.1155/2015/268796

**Published:** 2015-06-08

**Authors:** Andrea Perdicchi, Giacomo Peluso, Daniela Iacovello, Marco Balestrieri, Martina Delle Fave, Solmaz Abdolrahimzadeh, Gian Luca Scuderi, Vito Fenicia, Santi Maria Recupero

**Affiliations:** ^1^Ophthalmology Unit, NESMOS Department, Sant'Andrea Hospital, Faculty of Medicine and Psychology, “Sapienza” University of Rome, Via di Grottarossa 1035-1039, 00189 Rome, Italy; ^2^Ophthalmology Unit, Azienda Policlinico Umberto I, “Sapienza” University of Rome, Viale del Policlinico 155, 00186 Rome, Italy

## Abstract

*Purpose*. To detect the effects of intravitreal ranibizumab injections on GCC in patients with wet AMD. *Methods*. 32 wet AMD eyes were selected and submitted at three ranibizumab injections. RTVue-OCT GCC and MM5 protocol were performed before treatment and twenty days after each injection. *Results*. At baseline mean GCC thickness was 93.9 ± 18.5 *μ*m. Twenty days after each intravitreal injection it was, respectively, 85.8 ± 10.1, 86.5 ± 9.3, and 91.1 ± 11.5 *μ*m, without statistical significance. A significant improvement in visual acuity (*P* = 0.031) and a reduction of mean foveal (*P* = 0.001) and macular thickness (*P* = 0.001) were observed. *Conclusion*. The clinical results confirm therapeutic efficacy of intravitreal injections of ranibizumab in wet AMD. A contemporary not statistically significant reduction of GCC thickness suggests that the loading phase of ranibizumab does not have any toxic effects on ganglion cell complex.

## 1. Introduction

Age-related macular degeneration (AMD) is the most common cause of severe vision deficiency in elderly people in industrialized countries [1]. Wet or neovascular AMD is characterized by growth of new blood vessels caused by an abnormal release of the vascular endothelial growth factor-A (VEGF-A). This process leads to intra- and subretinal hemorrhagic and/or exudative alterations, with a consequent hemorrhagic detachment of the macular area and subsequent degeneration scar with central vision loss in varying amounts depending on the case [2]. The use of antiangiogenic drugs (anti-VEGF-A antibodies), administered by intravitreal injection, is one of the most effective treatments of neovascular AMD [3]. Recently many antiangiogenic and steroid drugs have been proposed in the treatment of exudative AMD and other rare retinal diseases and among these, ranibizumab in particular, administered by intravitreal injections, was mainly used in the treatment of exudative AMD [4–10]. Usually a sequence of three intravitreal injections of ranibizumab, at one-month intervals, represents the first dose or so-called “loading phase” of therapy in exudative AMD [11]. Additional intravitreal injections may be administered as needed according to the clinical progress of maculopathy and data provided by optical coherence tomography (OCT) and fluorescein angiography (FAG).

It should be considered that VEGF-A is also an important neurotrophic factor involved in the central nervous system, for the development of many cell types, including glial and neural cells [12]. Therefore, if on one hand the administration of anti-VEGF-A drugs inhibits chorioretinal neoangiogenesis that is present in wet AMD, on the other it may also inhibit the neuroprotective function of VEGF-A, with toxic effects on the retinal nerve structures. The macula region contains over 50% of all retinal ganglion cells and is probably the ideal region to detect early cell loss and changes over time because of the high density of cells. The function and structure of the retinal ganglion cells complex (GCC) encompass three layers in the inner retina: (1) the retinal nerve fiber layer (RNFL) which is made up of the ganglion cell axons, (2) the ganglion cell layer (GCL) which is made up of the ganglion cell bodies, and (3) the inner-plexiform layer (IPL) which is made up of the ganglion cell dendrites. It is also essential to realize that GCC analysis is not limited to the differential diagnosis and management of glaucoma but also is appliable in numerous neurological and retinal conditions.

The aim of our study was to detect the potential alteration and toxicity induced by the loading phase of therapy with ranibizumab (three intravitreal injections in three months) on ganglion cell complex (GCC) thickness measured by OCT in patients with wet AMD.

## 2. Methods 

32 eyes of 32 patients, 16 men and 16 women, aged between 75 and 95 years (mean age ± SD, 79.4 ± 7.1 years) diagnosed with wet AMD, were selected. Inclusion criteria were best visual acuity better than 0.7 logMAR units at Snellen optotype in the eye affected by neovascular AMD, intraocular pressure (IOP) less than 20 mmHg, without current or past topic hypotensive therapy, and ultrasonic pachymetry between 520 and 580 *μ*m. None of the patients had been previously treated with intravitreal injections. Exclusion criteria included retinal diseases responsible for maculopathy such as diabetic and/or hypertensive retinopathy or thrombosis of the retinal central vein. Other exclusion criteria were advanced cataract, anterior and/or posterior uveitis, optic neuritis, or dioptric means opacity that would make an OCT examination unreliable. According to the declaration of Helsinki, at the time of recruitment, informed consent to participate in the study was read and signed by all patients.

A complete ocular examination was performed on all patients within the week before the first intravitreal injection of ranibizumab, with the measurement of best corrected visual acuity by the ETDRS system and calculated in logMAR units, biomicroscopy of the anterior and posterior segments by slit lamp, examination of the ocular fundus with binocular indirect ophthalmoscopy, and evaluation of the retinal vascularization by fluorescein angiography (FAG). Intraocular pressure (IOP) was measured by Goldmann applanation tonometer and ICare tonometer [13].

**Figure 1:**
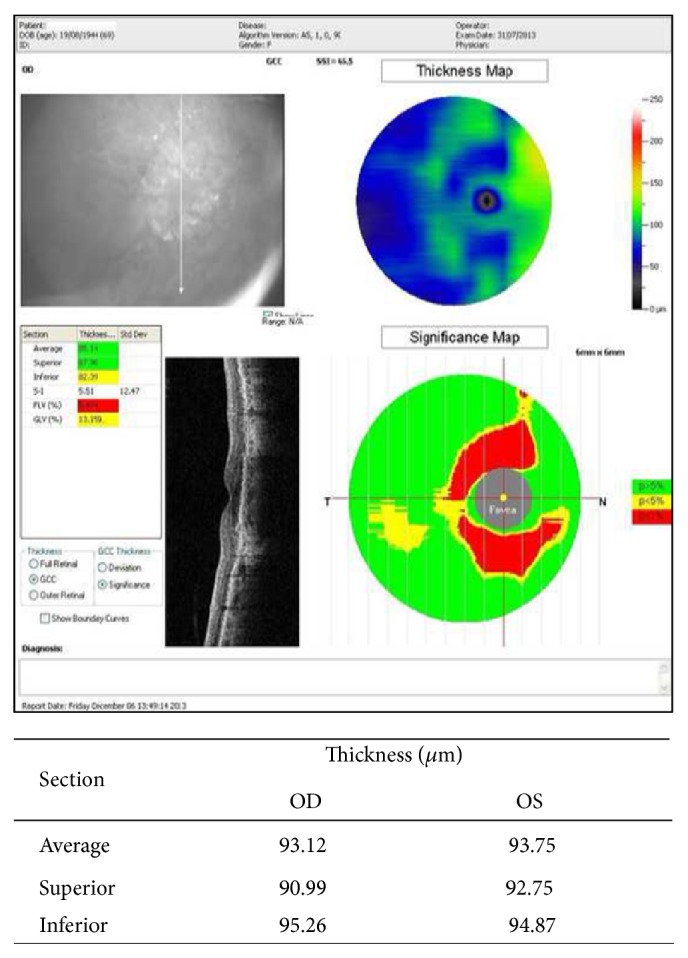
Thickness map, significance map, and average value of GCC.

Selected eyes were subsequently subjected to a cycle of three intravitreal injections of 0.5 mg of ranibizumab in a volume of 0.05 mL monthly. An OCT examination was performed the week before and twenty days after each injection. Ganglion cell complex (GCC) thickness was evaluated by GCC protocol of RTVue-OCT instrument (OPTOVUE), to assess the average value expressed in millimicrons (*μ*m) of three layers constituting GCC (nerve fiber layer, ganglion cell layer, and inner plexiform layer) [14, 15], (Figure 1).

**Figure 2:**
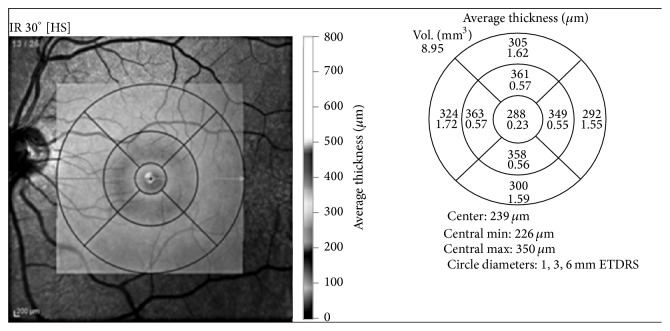
Example of macular map, divided into 9 areas.

A macular map of 5 mm (MM5) protocol of RTVue-OCT was used to assess foveal and macular thickness (Figure 2). A numerical value, expressed in millimicrons (*μ*m), placed at the center of the scanning macular map is the foveal thickness. Macular thickness was calculated by summing the values of thickness of each of the 9 map areas provided by OCT (central, inner/outer superior, inner/outer inferior, inner/outer nasal, and inner/outer temporal) and divided by the same number of areas.

Macular, foveal, and GCC thickness were evaluated at every control performed within 20 days after each injection of ranibizumab. All RTVue-OCT parameters were compared with the baseline parameters of each patient (Table 1). Intraocular pressure (IOP) was measured after each injection of ranibizumab at 3 times: an hour after treatment and the day after and a third one at the control performed twenty days after the intravitreal injection.

## 3. Statistical Analysis

Student's *t*-test for independent data and for paired data was used to compare parameters assessed before and after each intravitreal injection. All quantitative values were expressed as mean and standard deviations (SDs). *P* value lower than 0.05 was considered statistically significant. Confidence interval was calculated at 95%. All tests were performed by SPSS software (Statistical Package for Social Science, 9th version) for Windows.

## 4. Results

In affected eyes mean visual acuity before treatment (baseline) was 0.34 ± 0.22 logMAR units. An average increase of −0.05 ± 0.06 logMAR units (*P* = 0.019) was observed 20 days after the first intravitreal (IVT) injection of ranibizumab, An average increase of −0.04 ± 0.17 logMAR units (*P* = 0.004) was observed 20 days after the second injection, and a steady mean visual acuity of 0.22 ± 0.27 logMAR units (*P* = 0.031) was observed 20 days after the third injection (Table 2).

Assessed mean ganglion cell complex (GCC) thickness was 93.9 ± 18.5 *μ*m before the first injection (baseline). At a distance of 20 days after each intravitreal (IVT) injection, it was, respectively, 85.8 ± 10.1, 86.5 ± 9.3, and 91.1 ± 11.5 *μ*m with an average reduction of 8.1 ± 20.2 *μ*m (*P* = 0.123) after the 1st injection, 7.3 ± 22.3 *μ*m (*P* = 0.193) after the 2nd, and 2.7 ± 19.6 *μ*m (*P* = 0.577) after the 3rd (Table 3).

Assessed mean foveal thickness was 354.3 ± 61.9 *μ*m before the first intravitreal injection (baseline). Foveal thickness was significantly reduced after each intravitreal (IVT) injection: 295.8 ± 56.6 *μ*m after the first injection (mean reduction from baseline 58.5 ± 74.6 *μ*m, *P* = 0.005), 290.2 ± 60.9 *μ*m after the second injection (mean reduction from baseline 64.1 ± 82.6 *μ*m, *P* = 0.006), and 290.8 ± 43.9 *μ*m (mean reduction from baseline 63.4 ± 66.6 *μ*m, *P* = 0.001) after the third (Table 4).

Assessed mean macular thickness was 326.8 ± 30.6 *μ*m before the first injection (baseline). Also macular thickness was significantly reduced after each intravitreal (IVT) injection: 289.7 ± 28.6 *μ*m after the first injection (mean reduction from baseline 37.1 ± 32.3 *μ*m, *P* < 0.05), 282.8 ± 28.5 *μ*m after the second injection (mean reduction from baseline 39.2 ± 30.2 *μ*m, *P* < 0.05), and 291.3 ± 27.1 *μ*m (mean reduction from baseline 32.8 ± 22.9 *μ*m, *P* < 0.05) after the third (Table 5).

Assessed mean macular outer layer thickness was 201.9 ± 30.8 *μ*m before the first injection (baseline). Also macular outer layer thickness was significantly reduced after each intravitreal (IVT) injection: 184.3 ± 24.6 *μ*m after the first injection (mean reduction from baseline 17.6 ± 7.3 *μ*m, *P* < 0.05), 176.6 ± 26.4 *μ*m after the second injection (mean reduction from baseline 25.3 ± 7.2 *μ*m, *P* < 0.05), and 183.1 ± 22.5 *μ*m (mean reduction from baseline 18.8 ± 8.9 *μ*m, *P* < 0.05) after the third (Table 6).

Mean intraocular pressure (IOP) of wet AMD-affected eyes, measured with the two different instruments, at baseline was 15 ± 1.9 mmHg. A statistically significant IOP increase was not recorded after each intravitreal (IVT) injection of ranibizumab in any patient: mean IOP variation was 0.4 ± 1.9 mmHg (*P* = 0.450) after the first injection, 1.0 ± 2.3 mmHg (*P* = 0.127) after the second, and 0.7 ± 2.01 mmHg (*P* = 0.181) after the third (Table 7).

## 5. Discussion 

VEGF-A is known as being directly responsible for wet-form AMD chorioretinal neoangiogenesis, but it also plays a neuroprotective function in retinal nervous structures such as ganglion cell layer (GCC) and nerve fiber layer (RNFL), interacting especially with VEGFR2-receptor that is largely expressed over neuroretina [16–18]. It is observed that retinal nerve structures change in rats treated with six intravitreal injections of ranibizumab at weekly intervals. Progressive retinal ganglion cells degeneration, following injections, was recorded by assessing photopic full-field and focal-macular electroretinogram (ERG) reduced response [19]. In humans, retinal nerve fiber layer (RNFL) thickness reduction, evaluated by optical coherence tomography (OCT), was recorded after several intravitreal injections of ranibizumab. Nevertheless they do not change human macular and peripheral ganglion cells function, with no alterations of full-field and focal-macular ERG intensity response observed [20, 21]. According to this scientific evidence and having today a specific software of RTVue-OCT for ganglion cell complex (GCC) thickness and near retinal layers examination, the purpose of this study was to evaluate GCC modifications assessed by OCT, after 3 intravitreal injections of ranibizumab administered monthly (loading therapy in exudative AMD), irrespective of any anatomical and functional improvement detected. GCC modifications might strictly depend on ranibizumab toxic action due to inhibition of the neuroprotective function of VEGF-A or on a temporary IOP increase caused by intravitreal injection. In fact it is known that IOP increase resulting from intraocular volume increase, and following GCC ischemic events, is a possible complication of an intravitreal injection of ranibizumab [22–24]. Nevertheless, it is observed that it occurs only a few minutes following the injection, with intraocular pressure normalization within 30 minutes, without therapy [25, 26]. This study, in which IOP values appear stable one hour after the injection of ranibizumab and 24 hours after and 20 days following the treatment without a statistically significant mean IOP change (0.4 ± 1.9, *P* = 0.450 after the first injection; 1.0 ± 2.3, *P* = 0.127 after the second; and 0.7 ± 2.01, *P* = 0.181 after the third), confirms these results. In this study the OCT examination shows that GCC thickness slightly decreases from baseline values 20 days after ranibizumab intravitreal administration, especially after the first of the three intravitreal injections, although this variation does not appear statistically meaningful. The results of our study that for the first time provide a quantification of GCC thickness changes after the loading phase of ranibizumab confirm a slight GCC anatomical reduction that is nevertheless not statistically significant, resulting from the direct action of ranibizumab and not from an intraocular pressure increase following the injection. According to other authors, GCC reduction observed after intravitreal ranibizumab may result from a progressive arteriolar vasoconstriction, a consequent retinal ischemia associated with glutamate release that may damage ganglion cells, particularly sensitive to this substance [27–29]. The lack of significant anatomical GCC decrease after the first 3 intravitreal injections of ranibizumab suggests the acceptable safety of this therapy (GCC average reduction: 8.1 ± 20.2, *P* = 0.123 after the first injection; 7.3 ± 22.3, *P* = 0.193 after the second; and 2.7 ± 19.6, *P* = 0.577 after the third). In addition ranibizumab efficacy in reducing macular edema, induced by typical wet-form AMD choroidal neovascularization, is also confirmed by foveal and macular thickness decrease from baseline values, assessed by OCT MM5 protocol (resp., *P* = 0.005 and *P* = 0.001 after the first injection, *P* = 0.006 and *P* = 0.001 after the second, and *P* = 0.001 and *P* = 0.001 after the third). In addition the significant reduction of the outer retinal layer thickness after the loading phase of ranibizumab (*P* = 0.001 after each injection versus baseline) confirms that the pathological retinal changes in typical wet-form AMD are mainly localized in this layer while the GCC is on the contrary localized in the inner retinal layer.

## 6. Conclusion

Statistically significant reduction of macular edema and improvement in mean visual acuity, associated with stable intraocular pressure, are fairly certain evidence of the clinical efficacy of the treatment with ranibizumab.

Today GCC thickness changes in patients who require intravitreal injections of ranibizumab following loading-phase therapy are unknown. It is not yet known whether additional intravitreal injections of anti-VEGF-A can progressively affect the ganglion cell thickness and function, and this will be the subject of future study [30, 31].

## Figures and Tables

**Table 1:** Baseline parameters.

Baseline	Eyes (*n* = 32)
Age (years)	79.4 ± 7.1
Sex M/F	16/16
Visual acuity (logMAR units)	0.34 ± 0.22
IOP (mmHg)	15 ± 1.9
Foveal thickness (*μ*m)	354.3 ± 61.9
Macular thickness (*μ*m)	326.8 ± 30.6
GCC thickness (*μ*m)	93.9 ± 18.5

**Table 2:** Mean visual acuity (log⁡MAR units).

	Cases (*n* = 32)	Difference from baseline	*P* value
Baseline	0.34 ± 0.22		
1° IVT	0.26 ± 0.23	−0.05 ± 0.06	(*P*) = 0.019
2° IVT	0.20 ± 0.23	−0.04 ± 0.17	(*P*) = 0.004
3° IVT	0.22 ± 0.27	−0.26 ± 0.23	(*P*) = 0.031

**Table 3:** Mean GCC thickness (*μ*m).

	Cases (*n* = 32)	*P* value	95% CI
Baseline	93.9 ± 18.5		
1° IVT	85.8 ± 10.1	(*P*) = 0.123	−2.41; 18.46
2° IVT	86.5 ± 9.3	(*P*) = 0.193	−4.13; 18.88
3° IVT	91.1 ± 11.5	(*P*) = 0.577	−7.39; 12.83

**Table 4:** Mean foveal thickness (*μ*m).

	Cases (*n* = 32)	*P* value	95% CI
Baseline	354.3 ± 61.9		
1° IVT	295.8 ± 56.6	(*P*) = 0.005	20.14; 96.91
2° IVT	290.2 ± 60.9	(*P*) = 0.006	21.63; 106.59
3° IVT	290.8 ± 43.9	(*P*) = 0.001	29.18; 97.75

**Table 5:** Mean macular thickness (*μ*m).

	Cases (*n* = 32)	*P* value	95% CI
Baseline	326.8 ± 30.6		
1° IVT	289.7 ± 28.6	(*P*) = 0.001	20.42; 53.74
2° IVT	282.8 ± 28.5	(*P*) = 0.001	23.08; 55.31
3° IVT	291.3 ± 27.1	(*P*) = 0.001	20.11; 45.49

**Table 6:** Mean macular outer layer thickness (*μ*m).

	Cases (*n* = 32)	*P* value	95% CI
Baseline	201.9 ± 30.8		
1° IVT	184.3 ± 24.6	(*P*) = 0.001	13.28; 41.90
2° IVT	176.6 ± 26.4	(*P*) = 0.001	15.92; 46.66
3° IVT	183.1 ± 22.5	(*P*) = 0.001	5.58; 31.97

**Table 7:** Mean intraocular pressure (mmHg).

	Cases (*n* = 32)	*P* value	95% CI
Baseline	15 ± 1.9		
1° IVT	15 ± 1.7	(*P*) = 0.450	−0.70; 1.50
2° IVT	14 ± 1.3	(*P*) = 0.127	−0.32; 2.32
3° IVT	15 ± 2.1	(*P*) = 0.181	−0.38; 1.85
